# Positive regulators of T cell functions as predictors of prognosis and microenvironment characteristics of low-grade gliomas

**DOI:** 10.3389/fimmu.2022.1089792

**Published:** 2023-01-16

**Authors:** Yang Li, Yabo Feng, Fushu Luo, Gang Peng, Yueran Li

**Affiliations:** ^1^ Department of Laboratory Medicine, Third Xiangya Hospital, Central South University, Changsha, Hunan, China; ^2^ Department of Neurosurgery, Xiangya Hospital, Central South University, Changsha, Hunan, China; ^3^ PET-CT Center, Chenzhou First People’s Hospital, Chenzhou, Hunan, China; ^4^ Department of Obstetrics and Gynecology, The Third Xiangya Hospital of Central South University, Changsha, Hunan, China

**Keywords:** low-grade glioma, positive regulators of T cell functions, tumor microenvironment, T cell function, immunotherapy

## Abstract

**Background:**

Low-grade gliomas (LGG) are one of the most prevalent types of brain cancers. The efficacy of immunotherapy in LGG is limited compared to other cancers. Immunosuppression in the tumor microenvironment (TME) of LGG is one of the main reasons for the low efficacy of immunotherapy. Recent studies have identified 33 positive regulators of T cell functions (TPRs) that play a critical role in promoting the proliferation, activity, and functions of multiple immunocytes. However, their role in the TME of LGG has not been investigated. This study aimed to construct a risk model based on these TPRs and to detect the significance of immunotypes in predicting LGG prognosis and immunotherapy efficacy.

**Methods:**

A total of 688 LGGs and 202 normal brain tissues were extracted from The Cancer Genome Atlas (TCGA), Chinese Glioma Genome Atlas (CGGA), and Genotype-Tissue Expression (GTEx) databases. The NMF R package was used to identify TRP-related subtypes. The TPR prognostic model was established using the least absolute shrinkage and selection operator (LASSO) algorithm to predict the overall survival of LGG samples.

**Results:**

The Subtype 2 patients had worse survival outcomes, suppressed immune function, and higher immune cell infiltration. A risk regression model consisting of 14 TPRs was established, and its performance was validated in CGGA325 cohorts. The low-risk group exhibited better overall survival, immune microenvironment, and immunotherapy response, as determined *via* the TIDE algorithm, indicating that increasing the level of immune infiltration can effectively improve the response to immunotherapy in the low-risk group. The risk score was determined to be an independent hazard factor (p<0.001) although other clinical features (age, sex, grade, IDH status, 1p19q codel status, MGMT status, and accepted radiotherapy) were considered. Lastly, high-risk groups in both cohorts revealed optimal drug responses to rapamycin, paclitaxel, JW-7-52-1, and bortezomib.

**Conclusions:**

Our study identified two distinct TPR subtypes and built a TPR signature to elucidate the characteristics of T cell proliferation in LGG and its association with immune status and prognosis. These findings shed light on possible immunotherapeutic strategies for LGGs.

## 1 Introduction

Gliomas account for approximately 81% of primary brain tumors (1). According to the fifth edition of the WHO Classification of Tumors of the Central Nervous System, gliomas are classified into low-grade (WHO grades I and II) and high-grade gliomas (WHO grades III and IV) based on their molecular biomarkers (2). Low-grade gliomas (LGG) usually grow relatively slowly and extensively infiltrate the surrounding brain tissue, making it impossible to fully eradicate (3). Surgery followed by radiotherapy and chemotherapy remain to be the primary treatment for LGG. However, even after treatment, low-grade gliomas eventually recur and progress to high-grade gliomas and even glioblastoma, which is the most aggressive and deadly glioma type, with a median survival of 12–15 months after the final diagnosis (4, 5). Previous studies have identified multiple biomarkers related to the progression and prognosis of LGG. For instance, the presence of a 1p/19q deletion and IDH1 mutation conferred an improved prognosis in patients with LGG (6, 7). The IDH1 mutation also plays an important role in reprograming the phenotypic and functional diversity of myeloid cells in glioma TME (8). However, none of these genes have been applied to the clinical therapy of LGG. Furthermore, the mechanisms of tumorigenesis and invasion of LGG are still unknown. Thus, the identification of novel prognostic and therapeutic biomarkers that are involved in the tumorigenesis and progression of LGG is urgently needed.

The tumor microenvironment (TME) comprises the ecosystem surrounding the LGG, including immune cells, neurons, blood vessels, extracellular matrix, stromal cells, and signaling molecules (9). Immunotherapy strategies that target molecules of the TME, including checkpoint blockade, adoptive cellular therapy, and cancer vaccinology, have been developed and proven to be effective in several cancers (10–12). For example, immune checkpoint blockade (ICB) of programmed death-1 (PD-1) and its ligand PD-L1 is the most effective treatment for LUAD by regulating T cell activation (13). However, immunotherapeutic modalities yield limited success in gliomas because of their profound immunosuppressive environment. Gliomas present numerous obstacles to immunotherapy, including immune cell dysfunction, myeloid dysfunction, and tumor-related immunosuppressive factors (14). Moreover, the existence of a blood-brain barrier makes the delivery of immune drugs into gliomas difficult (15, 16). Although checkpoint blockade drugs, such as CTLA-4 and PD-1 inhibitors, have shown robust responses and have been applied in clinical trials in multiple cancers, the efficacy of CTLA-4 and PD-1 inhibitors in the treatment of LGG is limited (16). Human monoclonal antibodies (mAbs) against the immune system response modulators CTLA-4 (ipilimumab) and programmed cell death-1 (PD-1) (pembrolizumab and nivolumab) have been reported to achieve a significant clinical benefit for multiple cancers, but also have low efficacy in the treatment of gliomas (17, 18).

A recent study showed that chimeric antigen receptor (CAR) therapy using engineered autologous T cells redirected against tumor antigens has achieved success in the treatment of blood cancers and has been approved for clinical application (19). However, the suppression of T cell effector functions in the TME of solid tumors makes the efficacy of CAR T cell therapy much lower than in blood cancers. Recently, Legut et al. screened 33 positive regulators of T cell functions (TPRs) based on a genome-scale screen (20). Research has also shown that TPRs can increase the proliferation and activation of primary human CD4^+^ and CD8^+^ T cells and their secretion of key cytokines (20). Furthermore, adaptive immune responses in cancers also rely on the antigen-specific activation of naive T cells and their coordinated signals, both of which are essential for T cell activation (21). However, the regulatory T cells (Tregs) in LGG notably secrete immunosuppressive cytokines and downmodulate co-stimulatory molecules to suppress effector T cell activation (14, 22). Thus, TPRs in LGG may increase the activation of CD4+ and CD8+ T cells and act as ideal targets for developing novel immunotherapeutic approaches.

Here, we aimed to construct a risk model based on TPRs and detect the significance of immunotypes in predicting LGG prognosis and immunotherapy efficacy. Our study highlights the interplay between TPRs and the TME and provides a potential therapeutic target for LGG.

## 2 Materials and methods

### 2.1 Whole data collection

The RNA sequencing data of 506 primary and recurrent LGG samples and 202 normal tissues with corresponding clinical phenotype files were downloaded from the Xena database (https://xenabrowser.net/datapages/, cohort: GETx). mRNA sequencing and relevant clinical information from 182 primary and recurrent LGG samples were acquired from the CGGA325 dataset of the Chinese Glioma Genome Atlas (CGGA) database (http://www.cgga.org.cn/index.jsp). Eighty TPRs were obtained from the Gene Set Enrichment Analysis (GSEA) database (https://www.gseamsigdb.org/gsea/msigdb/cards/GOBP_ACTIVATED_T_CELL_PROLIFERATION.html) and Legut et al.’s data (**Table S3**). SLC10A7 immunohistochemistry (IHC) validation was performed using the Human Protein Atlas (HPA) database (https://www.proteinatlas.org/). Single nucleotide variants (SNV), copy number variants, and methylation levels of TPRs were determined using the GSCA database (http://bioinfo.life.hust.edu.cn/GSCA/#/) (23).

### 2.2 Identified TPR subtypes

The NMF R package was utilized for non-negative matrix decomposition (NMF) clustering to recognize T-cell proliferation subtypes, and the optimal subtype number *K* was determined to be 2 based on the cophenetic value. NMF is an unsupervised clustering algorithm that extracts maximum differential clusters (24). Principal component analysis (PCA) was used to identify the reliability and robustness of TPR subtypes.

### 2.3 Identification of differentially expressed TPRs and functional enrichment analysis

The “limma” R package was utilized to perform differential expression analysis between two groups (LGG samples vs cerebellar hemisphere and cortex tissues) with a |log2 fold change|>1 and a false discovery rate (FDR) < 0.05. Functional enrichment analyses, including the Kyoto Encyclopedia of Genes and Genomes (KEGG), Gene Ontology (GO), and GSEA, were performed using the clusterProfiler R package with an FDR < 0.05.

### 2.4 Construction and efficacy evaluation of the prognostic TPR signature

Based on the DETs filtered above, univariate regression analysis was used to further minimize the number of DETs to 30. Then, the 30 DETs were involved in the minimal least absolute shrinkage and selection operator (LASSO) Cox regression analysis to identify the prognostic TPR signature using the glmnet and survival R package. The LASSO Cox regression algorithm was used to screen appropriate prognostic candidates and prevent model overfitting (25). Fourteen overall survival (OS) events were integrated into a multifactor regression Cox analysis to identify OS events. Based on this TPR-related gene signature, the risk score for each patient was calculated by adding the coefficient index and expression level of each TPR-related gene. Patients with LGG (TCGA-LGG and CGGA325 cohorts) were divided into high - and low-risk groups, based on their median risk score. The difference between the two groups was estimated using the survival R package. The Kaplan-Meier (KM) survival curve depicts the differences in the median survival time between the high-risk and low-risk groups. To assess the precise efficiency of the TPR signature, we applied the time-dependent receiver operating characteristic (tdROC) and area under the curve (AUC) at 1, 3, and 5 years using the timeROC R package. Univariate and multivariate regression Cox analyses were conducted with relative clinicopathological features to evaluate the potential independent prognostic value of the TRP signature. In addition, we performed a subgroup analysis of the clinical features between the high-risk and low-risk groups.

### 2.5 Pathway enrichment analysis in different groups

We applied enrichment analyses (GO/KEGG/GSEA) to explore the potential corresponding pathways based on differentially expressed genes between the high-risk and low-risk groups. Annotated gene sets (including “h.all.v7.5.1. symbols.gmt”, GO/KEGG *via* clusterProfier R package) were used as reference lists. The enrichment results are shown in **Tables S4, S5**.

### 2.6 TME and immunotherapy analysis

We compared the expression of immune regulators (chemokines, chemokine receptors, MHC, immune inhibitors, and immune stimulators *via* the TISDB database: http://cis.hku.hk/TISIDB/index.php) (Z-score) between the low-risk and high-risk groups. Then, we explored the immune cell infiltration *via* MCP-counter analysis (26) and the correlation between the ESTIMATE-related score (27) (immune scores, stromal scores, and tumor purity) and risk score using the IOBR package (28). The tumor immune dysfunction and exclusion (TIDE) algorithm can provide better predictive efficiency than other immune-related markers (including immune checkpoint genes and tumor mutation burden) for immune-related therapeutic efficacy (29). Hence, we further investigated the relationship between the risk score of each LGG patient and the TIDE score (http://tide.dfci.harvard.edu/) and analyzed the IPS score based on transcriptomic data from The Cancer Immunome Database (TCIA database: https://tcia.at/home).

### 2.7 Chemotherapeutic drug sensitivity analysis

To explore the potential application of the TPR signature and chemotherapeutic drug response, we calculated the predictive half-maximal inhibitory concentration (IC50) of all chemotherapeutic agents using the “pRRophetic” R package for both cohorts. Next, to further screen for better chemotherapeutic agents, we applied the calculated value *via* pRRophetic to predict the risk score with an AUC>0.8. Ultimately, the intersection in both cohorts was screened for four chemotherapeutic drugs.

### 2.8 Construction and validation of a nomogram

To analyze the clinical application of the TPR signature, we established the nomogram *via* the “rms” R package, including certain clinical features (Age, Gender, Grade, Radiotherapy information, 1p19q coding deletion, isocitrate dehydrogenase 1 (IDH) status, and MGMT status). The concordance index (C-index) was used to compare the predictive ability of the nomogram and clinical parameters. Calibration plots were constructed to evaluate the fitting efficiency between the predicted nomogram and actual OS. Decision curve analysis was used to assess the threshold expectation range of the nomogram in association with the clinical characteristics.

### 2.9 Cell culture and transfection

The LGG glioma cell lines (SHG-44 and HS683) were purchased from ATCC, and human glial cells (HEB) were obtained from the Cancer Center, Sun Yat-Sen University. All cell lines were cultured in Dulbecco’s modified Eagle’s medium (DMEM; HyClone, United States) supplemented with 10% fetal bovine serum at 37°C in a 5% CO_2_ incubator.

Small interfering RNAs (siRNAs) against target genes were synthesized by GenPharma (Suzhou, China). Cells were transfected using the Lipofectamine^®^ RNAiMAX Transfection Reagent (Invitrogen, Carlsbad, California, United States) according to the manufacturer’s instructions. The siRNA sequences are listed in **Table S1**.

### 2.10 RNA extraction and quantitative PCR

Total RNA was extracted from the cell lines using the TRIzol reagent (Invitrogen, Carlsbad, CA, United States) according to the manufacturer’s instructions. The extracted RNA was reverse-transcribed into complementary DNA using the PrimeScript™ RT Reagent Kit (Takara, Dalian, China). Real-time PCR was performed using the SYBR Green Real-Time PCR Kit (Takara, Dalian, China). β-Actin was used as an endogenous control. The fold changes in gene expression levels were calculated using the 2^-ΔΔCT^ method. The primer sequences are listed in **Table S2**.

### 2.11 Cell proliferation, migration, and crystal violet staining assays

SHG-44 and HS683 cells were seeded in 96-well plates at a density of 3000 cells/well. Cell viability was measured using the Cell Counting Kit-8 (Sigma-Aldrich, Shanghai, China) every 24 h, according to the manufacturer’s instructions. Transwell migration assays were performed using 24-well transwell chambers (Corning, NY, United States). SHG-44 and HS683 cells (5.0 × 10^4^) were suspended in 300 µL of serum-free medium and seeded into the upper chambers; then, 600 µL of medium containing 20% FBS was added to the lower chambers. After 23 h, the migratory cells were fixed with 4% paraformaldehyde (PFA) and stained with 1% crystal violet. A crystal violet staining assay was performed to detect the ability of SHG-44 and HS683 cells to form colonies. Cells (1000 cells/well) were seeded into 6-well plates and cultured for 10 days. The culture media in the wells were discarded, the cells were fixed with 4% PFA for 10 min and then stained with 1% crystal violet for 5 min. The cells were photographed after washing with DPSB thrice.

### 2.12 Statistical analysis

All statistical analyses were performed using the R software (version 4.0.3; https://www.R-project.org). The Wilcoxon test was applied to continuous variables, and Spearman’s method was used to estimate the correlation coefficient between the two groups. Clinicopathological data for patients with LGG grouped by TRP signature were analyzed using the chi-square test, and the log-rank test was used for survival analysis. All results were considered statistically significant at p < 0.05.

## 3 Results

### 3.1 The multi-omics analysis of TPRs

**Figure 1:**
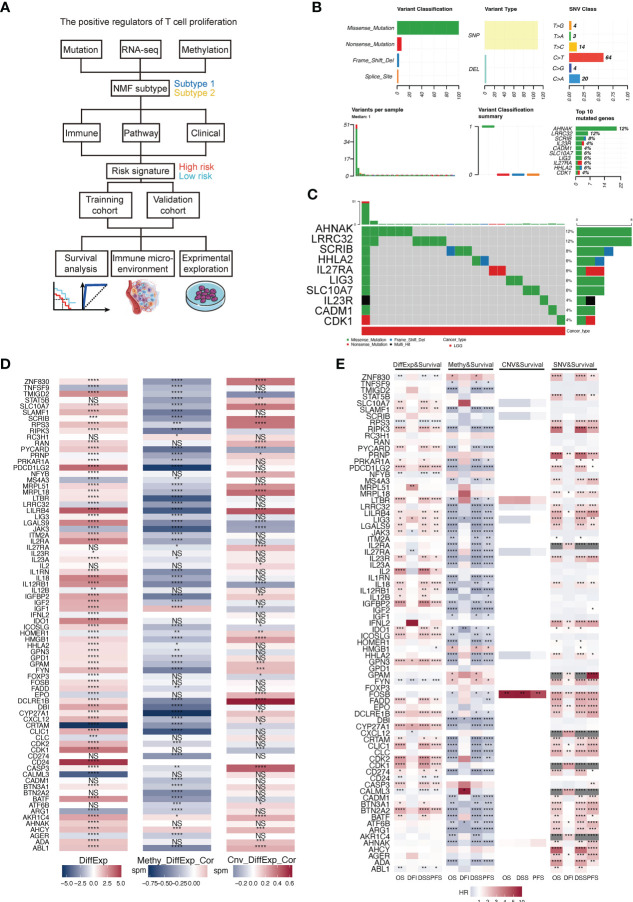
**(A)** Flowchart of this study. **(B)** A summary of the single nucleotide variants (SNV) of TPRs in TCGA-LGG cohorts. **(C)** A landscape of the genomic status of TPRs in TCGA-LGG cohorts. **(D)** The correlation between the differential expression of TPRs and methylation level/copy number aberration (CNA) status; DiffExp, log2 fold change of differential expression; Methy_DiffExp_Cor, the correlation between the methylation level of TPRs and TPR expression; Cnv_DiffExp_Cor, the correlation between CNA status and TPR expression. **(E)** The correlation among TPR expression and four survival estimate methods; OS, overall survival; DFI, disease-free interval; DSS, disease-specific survival; PFS, progress-free survival. *p<0.05, **p<0.01, ***p<0.001, ****p<0.0001, ns: no significant difference.

We included 506 LGG samples in the study, including 708 acquired from the TCGA and GTEx cohorts (506 tumor samples and 202 normal samples) and 182 from the CGGA325 cohort. The detailed clinical information of the included patients is presented in **Table 1**. A flowchart of this study is shown in **Figure 1A**. First, we estimated a single nucleotide polymorphism (SNP) summary for the TCGA-LGG cohort. As shown in **Figure 1B**, the most common mutations were missense mutations, and the most frequently mutated genes were *AHNAK, LRRC32, SCRIB, IL23R, CADM1, SLC10A7, LIG3, IL27RA, HHLA2*, and *CDK1*. The mutation type and number in TCGA-LGG patients are shown in **Figure 1C**. We then estimated the correlation between the mRNA expression of TPRs and genomic methylation. While a higher mRNA expression of TPRs was negatively correlated with methylation status, less TPR expression was positively correlated with copy number variation (CNV) status in LGG patients (**Figure 1D**). Ultimately, we evaluated patient survival based on mRNA expression, methylation, CNV, and single nucleotide variant (SNV) status. TPR expression and SNV status were regarded as risk factors for overall survival (OS), disease-specific survival (DSS), and progression-free survival (PFS). Furthermore, a lower TPR methylation status appeared to be a risk factor (**Figure 1E**).

**Table 1:** clinicopathological characteristics between high-risk and low-risk group in TCGA-LGG and CGGA325 cohorts.

	TCGA-LGG		CGGA325		
	High	Low	High	Low	p-value
	(N = 253)	(N = 250)	(N = 91)	(N = 91)	
Age					
<45	130 (51.4%)	132 (52.8%)	60 (65.9%)	69 (75.8%)	0.0167
>=45	94 (37.2%)	91 (36.4%)	31 (34.1%)	22 (24.2%)	
Missing	29 (11.5%)	27 (10.8%)	0 (0%)	0 (0%)	
Gender					
Female	105 (41.5%)	90 (36.0%)	33 (36.3%)	38 (41.8%)	0.304
Male	119 (47.0%)	133 (53.2%)	58 (63.7%)	53 (58.2%)	
Missing	29 (11.5%)	27 (10.8%)	0 (0%)	0 (0%)	
Grade					
II	102 (40.3%)	110 (44.0%)	31 (34.1%)	72 (79.1%)	<0.001
III	122 (48.2%)	113 (45.2%)	60 (65.9%)	19 (20.9%)	
Missing	29 (11.5%)	27 (10.8%)	0 (0%)	0 (0%)	
Radiotherapy					
No	85 (33.6%)	89 (35.6%)	17 (18.7%)	15 (16.5%)	<0.001
Yes	141 (55.7%)	143 (57.2%)	69 (75.8%)	73 (80.2%)	
Missing	27 (10.7%)	18 (7.2%)	5 (5.5%)	3 (3.3%)	
IDH_status					
Mutant	201 (79.4%)	205 (82.0%)	48 (52.7%)	85 (93.4%)	<0.001
Wildtype	51 (20.2%)	43 (17.2%)	43 (47.3%)	5 (5.5%)	
Missing	1 (0.4%)	2 (0.8%)	0 (0%)	1 (1.1%)	
1p19q					
Codel	73 (28.9%)	92 (36.8%)	12 (13.2%)	48 (52.7%)	<0.001
Non-codel	180 (71.1%)	158 (63.2%)	78 (85.7%)	42 (46.2%)	
Missing	0 (0%)	0 (0%)	1 (1.1%)	1 (1.1%)	
MGMT_status					
Methylated	201 (79.4%)	214 (85.6%)	39 (42.9%)	50 (54.9%)	<0.001
Unmethylated	52 (20.6%)	36 (14.4%)	41 (45.1%)	36 (39.6%)	
Missing	0 (0%)	0 (0%)	11 (12.1%)	5 (5.5%)	
Event					
Alive	154 (60.9%)	220 (88.0%)	26 (28.6%)	57 (62.6%)	<0.001
Death	99 (39.1%)	30 (12.0%)	61 (67.0%)	31 (34.1%)	
Missing	0 (0%)	0 (0%)	4 (4.4%)	3 (3.3%)	
Subtype					
Subtype 1	54 (21.3%)	122 (48.8%)	56 (61.5%)	91 (100%)	<0.001
Subtype 2	199 (78.7%)	128 (51.2%)	35 (38.5%)	0 (0%)	

### 3.2 Identification of T cell proliferation subtype

**Figure 2:**
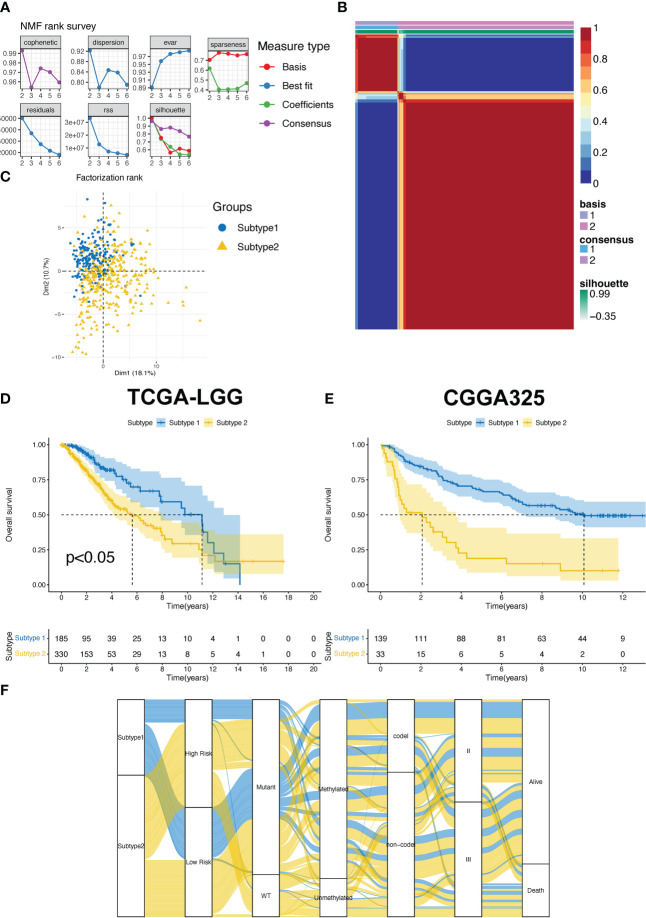
**(A)** The factorization rank diagram of the TPR subtype. **(B)** The clustering results using k=2 is shown for TCGA-LGG cohort. **(C)** Principal component analysis of the two subtypes in the TCGA-LGG cohort. **(D, E)** The overall survival rates of the two subtypes in the TCGA-LGG **(D)** and CGGA325 cohorts **(E)**. **(F)** The Sankey diagram exhibits the correlation between the subtype and clinical features.

The T cell proliferation status of patients with LGG is a critical factor for evaluating therapeutic efficacy. We used NMF R packages to identify the two subtypes of T cell proliferation. The classification of proliferating T cell subtypes was the most stable when *k*=2 (**Figures 2A, B**). Meanwhile, the results of the PCA revealed that subtype II patients accounted for more in the first, third, and fourth quadrants, whereas subtype I patients accounted for more in the second quadrant (**Figure 2C**). Compared with subtype 2, patients with subtype 1 had a longer median OS in the TCGA-LGG and CGGA325 cohorts (**Figures 2D, E**). In addition, we observed a correlation between the subtypes and other clinical features (**Figure 2F**).

To determine the crucial pathways and further explore the potential function of T cell proliferation between subtypes 1 and 2, we conducted a pathway enrichment analysis. First, we evaluated pathway enrichment results extracted from the HALLMARK gene set. The tissue-enriched genes (TEGs) of subtype 2 were the most correlated with the development of cancer (allograft rejection, coagulation, and epithelial-mesenchymal transition) and immune-related pathways (inflammatory response, interferon alpha/gamma response, and TNF signaling *via* NFκB; **Figure S1A**) as opposed to those of subtype 1. GO and KEGG pathway analyses were then performed using the GO terms biological process (BP), molecular function (MF), cellular component (CC), and KEGG. Ultimately, the top 15 characteristics of enrichment results with adjusted p-value < 0.05 were depicted in **Figures S2B-E**. The KEGG enrichment results revealed that patients with subtype 2 were mainly correlated with cancer-related pathways, including the MAPK signaling pathway, calcium signaling pathway, and Ras signaling pathway (**Figure S1B**). The GO enrichment results indicated that subtype 2 was mainly related to the regulation of hormone levels (**Figures S1C, D**) and DNA-binding-related regulation (**Figure S1E**).

**Figure 3:**
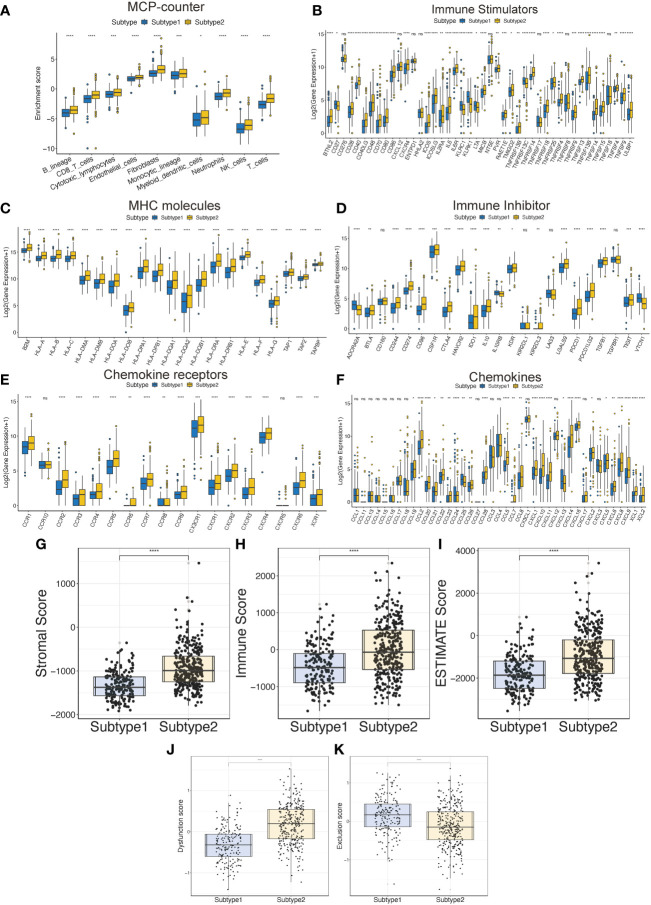
The tumor immune microenvironment characteristics between the TPR subtypes. **(A)** The immune cell infiltration was assessed *via* the MCP-counter algorithm in the TCGA-LGG cohort. **(B-F)** The expression of immune-related genes between the two subtypes in the TCGA-LGG cohort, including immune stimulators **(B)**, MHC molecules **(C)**, immune inhibitors **(D)**, chemokine receptors **(E)**, and chemokines **(F)**. **(G-I)** The ESTIMATE-related scores of the two subtypes in the TCGA-LGG cohort, including stromal score **(G)**, immune score **(H)**, and ESTIMATE score **(I)**. **(J, K)** T cell dysfunction and T cell exclusion scores between the two subtypes in the TCGA-LGG cohort were assessed *via* the ssGSEA algorithms. *p<0.05, **p<0.01, ***p<0.001, ****p<0.0001, ns: no significant difference.

To evaluate the differences in the tumor immune microenvironment between subtypes 2 and 1, we analyzed immune cell infiltration, expression of immune-related genes, and immune-related scores. First, the MCP-counter results demonstrated that the patients with subtype 2 had more T cells and other infiltrating immune cells compared to those with subtype 1 (**Figure 3A**). Subtype 2 had higher expression of immune stimulators, MHC molecules, immune inhibitors, chemokine receptors, and chemokines in the TCGA-LGG, compared to those in subtype 1 (**Figures 3B-F**). Similarly, higher stromal and immune scores and ESTIMATE scores indicated that patients with subtype 2 had more immune cell and stromal cell infiltration (**Figures 3G-I**). Hot tumors with more T cell infiltration and favorable survival have acquired better immunotherapy efficacy (30). Meanwhile, LGG patients are more immunologically quiet and have moderate lymphocyte depletion (31). However, subtype 2 showed more infiltration and a worse OS, suggesting that patients with this subtype had dysfunctional T-cells (**Figure 3J**). On the other hand, infiltrates in patients with subtype 1 excluded T-cells (**Figure 3K**).

### 3.3 Construction and efficacy evaluation of the prognostic TPR signature

To analyze the prognostic power of TPRs in OS, TEGs were added to the LASSO-multifactor Cox regression analysis, and the most corresponding prognostic TPR signature was filtered out. First, we identified 35 upregulated and eight downregulated TEGs in the heatmap and volcano plot (**Figures S2A, B**). The 30 genes were screened using univariate Cox regression analysis, with a significance of p <0.05. Ultimately, 30 TPRs were identified for the LASSO-Cox analysis.

**Figure 4:**
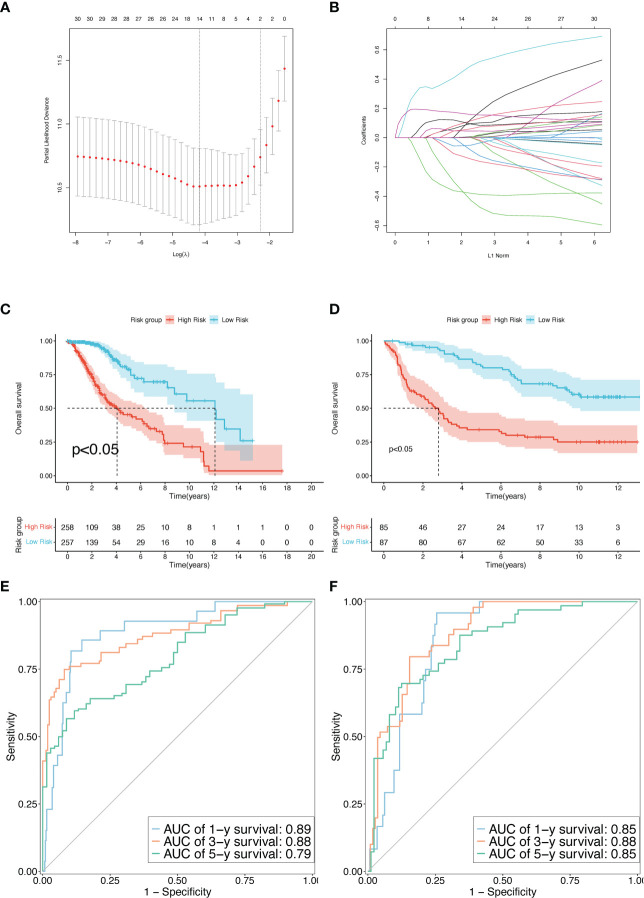
Construction and validation of the TPR signature. **(A)** The LASSO-Multi-Cox regression identified a beneficial signature *via* min lambda; left dash: min lambda, right dash: standard lambda. **(B)** Ten cross-validations for adjusting and optimizing the parameter screen out in the LASSO-Multi Cox regression. **(C, D)** The overall survival rate between high-risk and low**-**risk groups using the median risk score in the TCGA-LGG **(D)** and CGGA325 cohorts **(E)**. **(E, F)** Time-dependent receiver operating characteristic (tdROC) curves to determine the accuracy of the TPR signatures for predicting the mortality event of LGG patients in the TCGA-LGG **(D)** and CGGA325 cohorts **(E)**.

Based on the minimal lambda value that was selected through 10-fold cross-validations and 1000 iterations, 14 prognostic TRPs were screened (**Figures 4A, B**). The 14 TRP-related univariate Cox analysis data points are shown in **Table S6**. The risk score for each patient in the TCGA-LGG and CGGA325 cohorts was calculated using the following formula: Risk score = DBI*Coef_DBI_ + FYN*Coef_FYN_ + IL18*Coef_IL18_+ CDK1*Coef_CDK1_+ RPS3*Coef_RPS3_+ PDCD1LG2*Coef_PDCD1LG2_+ FADD*Coef_FADD_ + CXCL12*Coef_CXCL12_+ CLIC1*Coef_CLIC1_+ CDK2*Coef_CDK2_+ SLC10A7*Coef_SLC10A7_+ BATF*Coef_BATF_ + IGBP2*Coef_IGBP2_+ LRRC32*Coef_LRRC32_ (the sum of gene expression* coefficient index, **Table 2**). The patients with LGG in the high-risk group from the TCGA and CGGA325 cohorts had a worse prognosis than those in the low-risk group, based on the Kaplan-Meier survival analysis plot (p < 0.05, **Figures 4C, D**). To evaluate the prognostic power, tdROC analysis was applied at 1, 3, and 5 years in the TCGA-LGG and CGGA325 cohorts. The AUC exceeded 0.79 in both cohorts in all the different years, indicating an excellent prognostic recognition value (**Figures 4E, F**). These results indicate that the prognostic TPR signature and risk score algorithm may be regarded as a new classification system for LGG.

**Table 2:** The coefficient value of TRPs signature.

Genes	Coefficient
DBI	0.00263109
FYN	-0.3704738
IL18	0.08180692
CDK1	0.09880596
RPS3	-0.0555493
PDCD1LG2	0.04530573
FADD	0.07077506
CXCL12	-0.3712566
CLIC1	0.4546302
CDK2	0.10572267
SLC10A7	0.05215324
BATF	-0.04856
IGFBP2	0.16432256
LRRC32	-0.1291973

**Figure 5:**
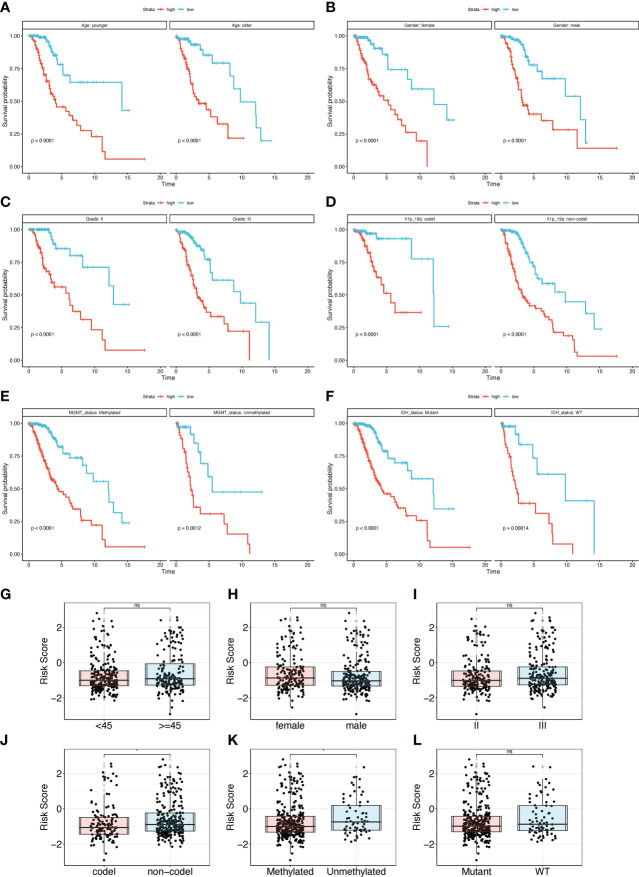
Subgroup analysis of the TPR signature for clinical features. **(A-F)** The overall survival rate of different subgroups in the TCGA-LGG cohort, including Age **(A)**, Gender **(B)**, Grade **(C)**, 1p19q status **(D)**, MGMT **(E)**, and IDH status **(F)**; 1p19q status: the coding and noncoding deletion status in the short arm of chromosome 1 and the long arm of chromosome 19; MGMT status: methylation status of *MGMT*; IDH status: *IDH1* mutant and wildtype. **(G-I)** The risk score between different clinical subgroup features in the TCGA-LGG cohort, including Age **(G)**, Gender **(H)**, Grade **(I)**, 1p19q **(J)**, MGMT **(K)**, and IDH status **(L)**.

Subgroup survival analyses were performed in the TCGA cohort to explore the correlation between the clinical features and the TPR signature of LGGs. As shown in **Figures 5A-F**, the median survival time of LGG patients in the high- and low-risk groups can also be distinguished. In addition, samples with MGMT unmethylation and a 1p19q non-coding deletion had higher risk scores than those with MGMT methylation and a 1p19q coding deletion (**Figures 5G-L**). Hence, the TPR signature can potentially be important in the development of LGGs.

Enrichment analysis was used to identify the critical pathways for cancer and to assess the potential function of the TPR signature between high- and low-risk samples. First, we identified significantly differentially expressed genes between the high- and low-risk groups. Then, the GO/KEGG/GSEA pathway analyses were conducted *via* the clusterProfield R package using the GO terms BP/MF/CC and KEGG. The top five characteristics of the enrichment results with an adjusted p-value < 0.05 are depicted in **Figure S2**. Pathway enrichment results indicated that the high-risk group was mainly correlated with immune-related pathways, including positive regulation of T cells, cytokine-cytokine receptor interaction, and lymphocyte-mediated immunity (**Figure S3A**). Subsequently, we evaluated the HALLMARK pathway enrichment results, which similarly revealed that high-risk groups had more immune-related pathway enrichment, including cytokine-cytokine receptor interaction, antigen processing and presentation, interferon inflammatory response, and interferon-gamma response (**Figures S3B-E**).

### 3.4 TME and immune-related response analysis

**Figure 6:**
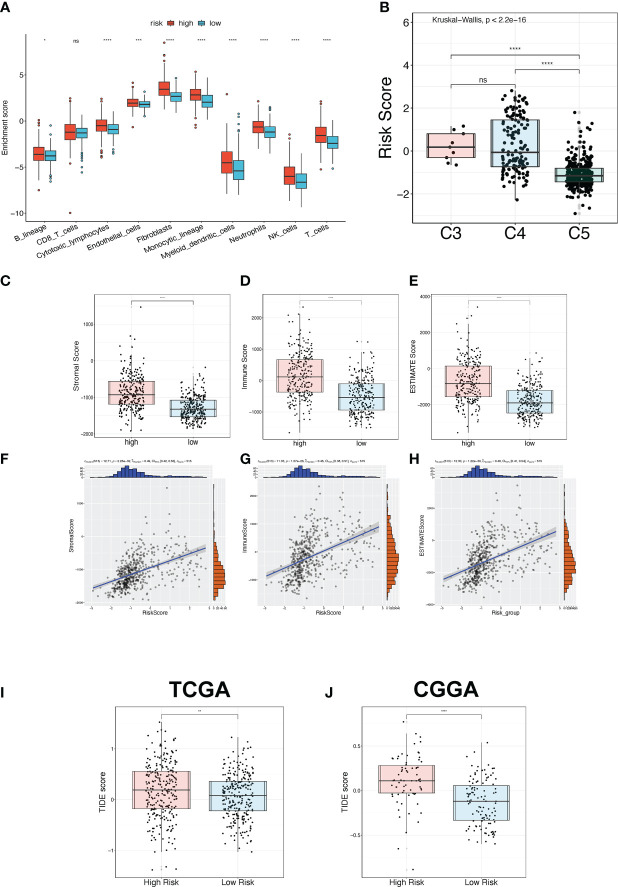
TME and immunotherapy prediction analysis. **(A)** The immune cell infiltration status between the high-risk and low-risk groups in the TCGA-LGG cohort was estimated *via* the MPC-counter algorithm. **(B)** The risk score between three immune subtypes. C3: Inflammatory; C4: moderate lymphocyte depletion; C5: immunologically quiet. **(C-E)** The ESTIMATE-related score between high-risk and low-risk groups in the TCGA-LGG cohort, including stromal score **(C)**, immune score **(D)**, and ESTIMATE score **(E)**. **(F-H)** The Pearson correlation analysis between the ESTIMATE-related score and risk score in the TCGA-LGG cohort, including stromal score **(F)**, immune score **(G)**, and ESTIMATE score **(H)**. **(I, J)** The TIDE score for predicting immunotherapy efficacy between the high-risk and low-risk groups in the TCGA-LGG **(I)** and CGGA325 **(J)** cohorts. *p<0.05, **p<0.01, ***p<0.001, ****p<0.0001, ns: no significant difference.

The relationship between the TPR signature and TME was elucidated in the TCGA-LGG and CGGA325 cohorts. First, we explored the expression of immune regulators in the high- and low-risk groups. Most of the expressed immune stimulators, MHC molecules, immune inhibitors, chemokine receptors, and chemokines were seen in the high-risk group (**Figures S4A-E**), indicating that the high-risk group may have more immune cell infiltration than the low-risk group. We then compared the TME of the high-risk and low-risk samples. Compared to the low-risk group, B lineage, cytotoxic lymphocytes, endothelial cells, fibroblasts, monocytic lineage, myeloid dendritic cells, neutrophils, NK cells, and T cells were more active in the high-risk group, as determined *via* the MCP-counter algorithm (**Figure 6A**). Thorsson et al. divided LGGs into the most immunologically quiet (C5) and moderately lymphocyte-depleted (C4) subgroups, which were conducted using 160 immune gene expression signatures (31). Patients with C5 features had better OS and lower risk scores (**Figure 6B**). Furthermore, based on the ESTIMATE algorithm, the ESTIMATE-related score (incorporating stromal, immune, and estimate scores) was significantly elevated in high-risk patients (**Figures 6C–E**). In addition, correlation analysis revealed that the stromal, immune, and estimated scores were positively correlated with risk scores of 0.49 (**Figure 6F**), 0.45 (**Figure 6G**), and 0.48 (**Figure 6H**), respectively. We used the TIDE algorithm to further determine the correlation between the risk score and immunotherapy (**Figures 6I, J**).

### 3.5 Screening for appropriate chemotherapeutic drugs

**Figure 7:**
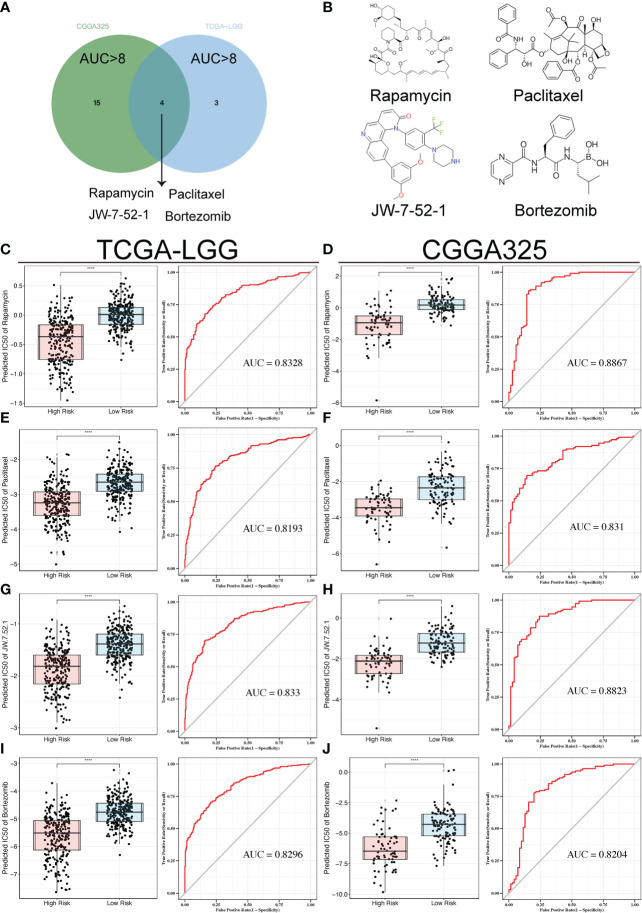
Drug sensitivity analyses in both cohorts. **(A)** Venn diagram showing the overlaps of the sensitivity to the top four drugs. **(B)** Tomographs of the structures of the four candidate drugs. **(C-J)** Box plots displaying the predicted IC50 of the four drugs in the high-risk and low-risk groups in the TCGA-LGG and CGGA325 cohorts, including Rapamycin **(C, D)**, Paclitaxel **(E, F)**, JW-7-52-1 **(G, H)**, and Bortezomib **(I, J)**; ROC analyses of the risk scores for the prediction of drug response. ****p<0.0001.

To discover the correlation between the TPR signature and drug response, we compared the chemotherapeutic response in high- and low-risk groups (p < 0.05 was considered significant) and determined the predictive efficiency for the risk score (threshold value: AUC > 0.8, p < 0.05). The responses to rapamycin, paclitaxel, JW-7-52-1, and bortezomib intersected in both cohorts (**Figures 7A, B**). The AUC values of all drugs are recorded in **Table S7**. We then compared the IC50 values of the drugs between the high- and low-risk groups in both cohorts. Patients in the low-risk group had a higher predictive IC50 value, indicating a worse response to rapamycin (**Figures 7C, D**), paclitaxel (**Figures 7E, F**), JW-7-52-1 (**Figures 7G, H**), and bortezomib (**Figures 7I, J**) than those in the high-risk group. These results also indicated that the patients with high-risk scores had a potential therapeutic response to the four drugs (**Figures 7C-J**).

### 3.6 Construction and validation of the Nomogram

**Figure 8:**
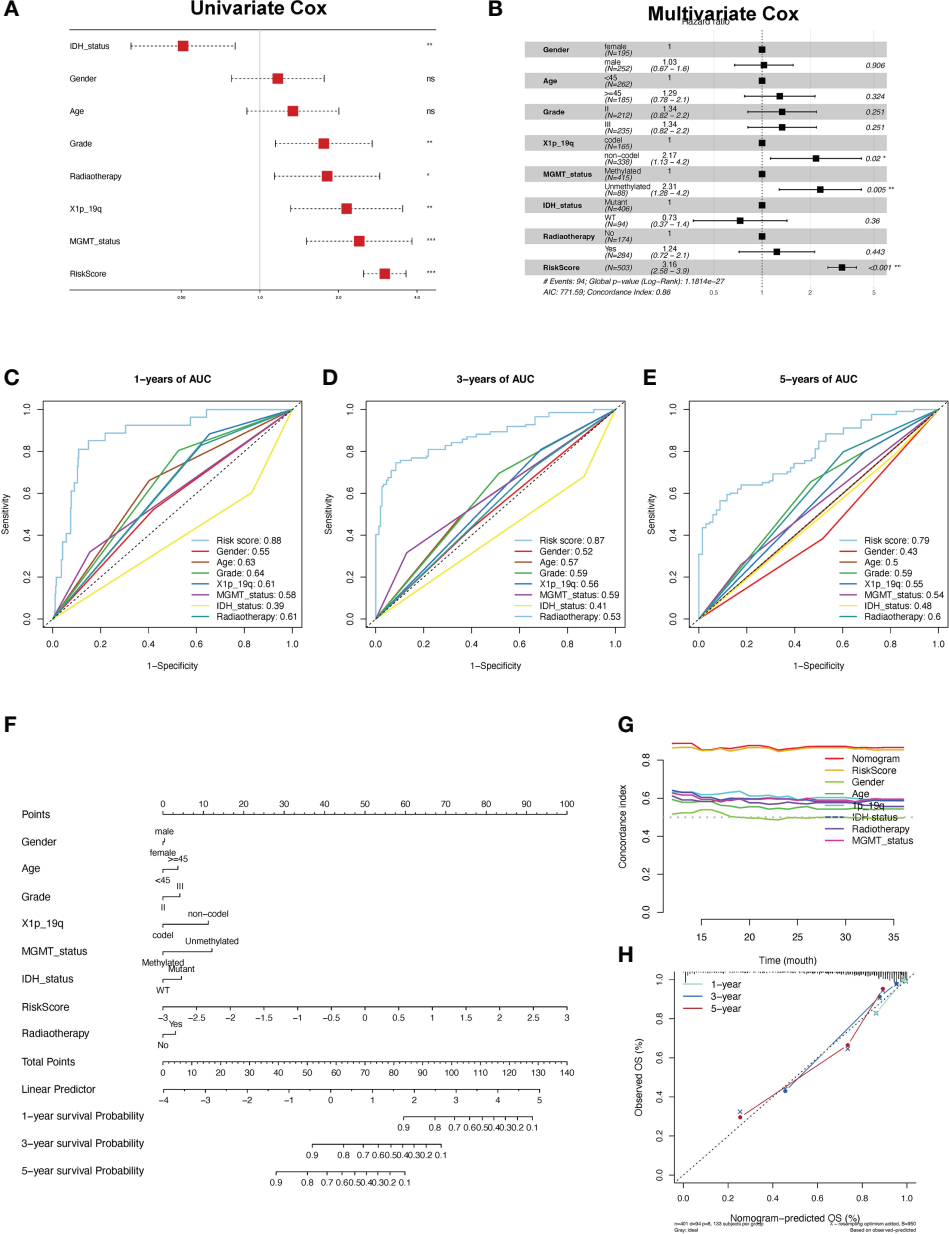
Construction and validation of the nomogram. **(A, B)** Univariate and multifactor Cox regression analysis between the risk scores and clinical features. **(C-E)** ROC analyses of the risk score for the prediction of overall survival status at 1 **(C)**, 3 **(D)**, and 5 years **(E)**. **(F)** Predicting the probabilities of survival *via* nomogram at 1, 3, and 5 years in the TCGA-LGG cohort. **(G)** The concordance index for predicting the probabilities for clinical features using the nomogram. **(H)** The calibration curve plots for predicting 1-, 3-, and 5-year OS in LGG patients in the TCGA cohort.

To further explore the clinical application of the risk score, we constructed a prognostic predictive nomogram. First, to evaluate the independent predictive ability of the risk score, we applied univariate and multivariate Cox regression analyses with relevant clinicopathological characteristics, including age, sex, grade, radiotherapy, 1p19q codel, IDH mutation, and MGMT status. The results of the multivariate regression Cox analysis indicated that high-risk scores, 1p19q non-codeletion genotypes, and unmethylated MGMT status were significantly related to a worse OS in LGG patients in the TCGA cohort (p < 0.001, **Figures 8A, B**). In addition, the depicted ROC curves illustrated that risk scores have better prognostic performance for OS prediction than other clinicopathological characteristics (**Figures 8C-E**). Based on the above clinicopathological characteristics, we constructed a nomogram score system to predict the 1-, 3-, and 5-year OS of patients with LGG (**Figure 8F**). Furthermore, the C-index results demonstrated that nomograms have a robust prognostic value compared with other clinicopathological features (**Figure 8G**). Subsequently, the findings of the calibration plot analysis revealed the reliability and applicability of the nomogram model based on the risk score and clinicopathological characteristics in real-world situations (**Figure 8H**).

### 3.7 SLC10A7 was upregulated in LGG and was crucial for the proliferation and migration of LGG cells

**Figure 9:**
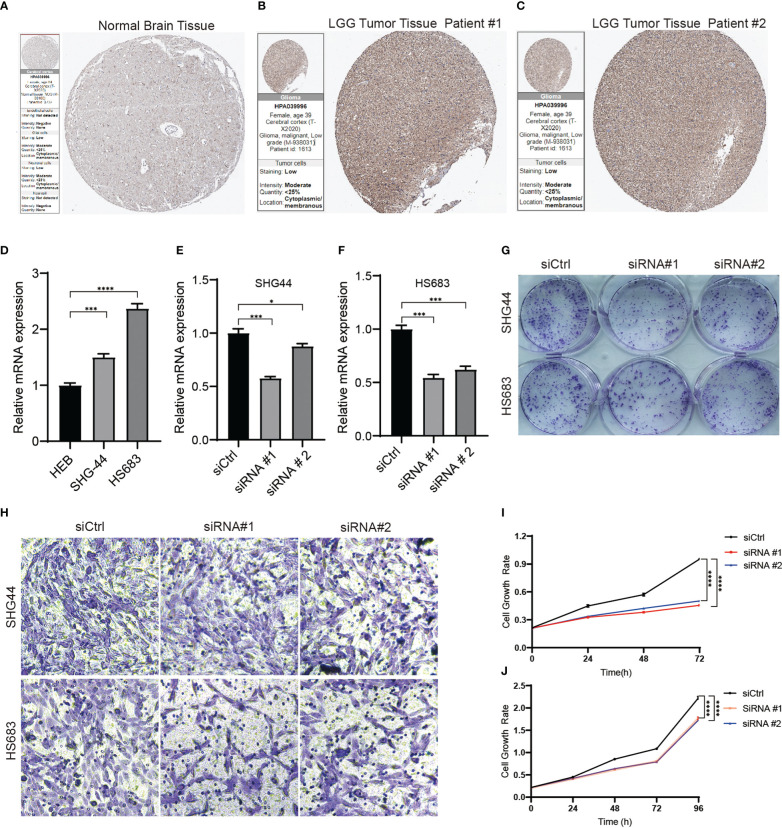
SLC10A7 was upregulated in LGG and was critical for the proliferation and migration of LGG cells. **(A-C)** SLC10A7 expression in normal brain tissue and LGG tumor tissue detected by immunochemistry. **(D)** The western blotting analysis confirmed that SLC10A7 was upregulated in LGG cell lines. **(E, F)** The efficacy of SLC10A7 knockdown in two LGG cell lines. **(G)** The colony formation ability was detected using the crystal violet staining assay. **(H)** Cell migration ability of the LGG cell lines transferred with control or SLC10A7 siRNA was examined *via* the Transwell assay. **(I, J)** Cell proliferation of the SHG-44 cell line **(I)** and HS683 cell line **(J)** transferred with control or SLC10A7 siRNA was detected *via* the CCK-8 assay. *P <0.1, ***P < 0.001, ****P < 0.0001.

SLC10A7 is one of the most highly expressed genes in LGG and is thus highly considered in the risk score. Immunohistochemistry data acquired from the Human Protein Atlas indicated that SCL10A7 was upregulated in LGG (**Figures 9A-C**). *In vitro* experiments also showed that SLC10A7 was upregulated in the LGG cell lines (**Figure 9D**). After knocking down SLC10A7 (**Figures 9E, F**), the colony formation ability, cell migration, and proliferation of LGG cell lines were decreased (**Figures 9G-J**).

## 4 Discussion

In recent decades, researchers have proven that the central nervous system has a unique lymphatic drainage system, which contributed to the development of immunotherapies for neuroinflammatory and neurodegenerative diseases and gliomas (32, 33). Immunotherapies, including immune-checkpoint inhibitors, cancer vaccines, and oncolytic viruses, have been investigated in patients with glioblastoma (GBM), but the treatment effects are limited (34). Despite these limits, immunotherapies may have promising therapeutic activity in LGGs by targeting alterations in their immune environment. Thus, it is important to determine the characteristics of the immune microenvironment of LGGs and identify novel prognostic markers that can act as therapeutic targets. In this study, we constructed a prognostic model based on TPRs and proved its feasibility for detecting LGG prognostic markers.

There are three reasons for immunosuppression in patients with LGG. The most critical reason is that regulatory T cells (Tregs) in LGG secrete immunosuppressive cytokines and downregulate the expression of stimulatory molecules to suppress effector T cell activation [13, 21]. The second reason is the reduced T-cell and myeloid cell infiltration in LGG (35). Lastly, the current standards of care for patients, including temozolomide chemotherapy, radiotherapy, and corticosteroids, also result in immunosuppressive effects in LGG (34). To overcome this immunosuppression, we constructed a prognostic model based on 43 selected differentially expressed TPRs. Patients with LGG can be divided into high- and low-risk groups based on a stratified model. We found that patients in the high-risk group had a worse prognosis than those in the low-risk group. The pathway enrichment analysis indicated that the high-risk group was mainly positively correlated with cytokine-cytokine receptor interaction, antigen processing and presentation, interferon inflammatory response, and interferon-gamma response, all of which are crucial for immune responses (36, 37). Furthermore, the expression of immune stimulators such as MHC molecules, immune inhibitors, chemokine receptors, and chemokines was upregulated in the high-risk group. Thus, we can conclude that the high-risk group has high immune cell infiltration and immune checkpoint activation, which results in a poor prognosis. This feature may have resulted from immunocyte recoding caused by the cytokines and chemokines in the LGG microenvironment. The recoded immunocytes would, in turn, promote the progression and invasion of LGGs (38, 39). The relatively higher rates of MGMT methylation and 1p19q co-deletion and a lower rate of IDH1 mutation also supported the notion that patients in the high-risk group had poor prognoses. In addition, we compared risk scores between the mutant group (*ATRX* and *TP53*) and the wild-type group. We further compared the OS probability between subgroups. As depicted in **Supplementary Figure 5**, there was no statistical difference in risk scores between the mutant group and the non-mutant group. Within the *ATRX* mutation subgroup, there was no statistically significant difference in OS between the high and low risk groups (cutoff: median value of risk score), indicating that the TRP signature may more suitable for prognostic assessment in patients with wild-type *ATRX*. Therefore, the prognostic model based on the 43 TPRs was able to predict the immune status and prognosis of patients with LGG.

Patients in the high-risk group have more immune and stromal cell infiltration, which can be called an immunosuppressed TME that includes macrophage-dominated and low lymphocytic infiltrates. Furthermore, the low-risk group had less immune cell infiltration, ESTIMATE-related score, and better median survival time than the high-risk group, indicating that these patients had an immune-quiet TME. These conclusions were consistent with the results of the immune cell infiltration and immune cell subtype analyses (**Figures 6A-G**). Thus, we can take advantage of the distinction between immunosuppressed and immune-quiet TME and perform individualized immunotherapy (30). To determine an optimal therapeutic strategy for the high-risk group, we selected rapamycin, paclitaxel, jw-7-52-1, and bortezomib for further research, all of which have been proven to be efficient in the treatment of GBM (40–43). After analyzing the IC50 values of the two groups, we concluded that the patients in the high-risk group had a better response to these drugs. Furthermore, to evaluate the clinical value of the risk score, we constructed a prognostic predictive nomogram and found that patients with high-risk scores had worse prognoses. The results of the multivariate regression Cox analysis also indicated that the risk scores had better prognostic performance in the prediction of OS in LGG when compared with the grade, radiotherapy, and 1p19q codel, IDH mutation, and MGMT statuses, all of which are clinicopathological characteristics related to the prognosis of LGG (44). Even though we took advantage of multiple platforms to analyze the immune microenvironment of the two groups, all of which were regarded as external verification*, in vitro* experiments are needed to verify the possibility of TPRs as novel targets for immunotherapy.

Based on a previous study, we identified 14 candidates that were associated with LGG. Most of these genes, including DBI (45), FYN (46), IL18 (47), CDK1 (48), RPS3 (49), PDCD1LG2 (50), FADD (51), CXCL12 (52), CLIC1 (53), CDK2 (54), IGFBP2 (55) and LRRC32 (56), have been reported to play critical roles in the development, stemness, and immunogenicity of gliomas. Compared to BATF, the SLC10A7 have the following features: higher expression with worse overall survival, lower methylation level, more missense mutation rate, and higher coefficient value in LGG (**Figures 1B-E** and **Table 2**). However, the exact role of SLC10A7 in LGG remains unknown. Multiple studies have demonstrated that SLC10A7 plays an important role in various human cancers (57, 58). Thus, we selected SLC10A7 for further research to determine its role in the development and invasion of LGG. SLC10A7 is an orphan member of the solute carrier (SLC) family 10 (SLC10), which encodes a 10-transmembrane-domain transporter located at the plasma membrane (59). SLC10A7 mutations are associated with skeletal dysplasia, amelogenesis imperfecta, and decreased bone mineral density (60). However, the exact molecular function of SLC10A7 in LGG development remains to be elucidated. In this study, we found that SLC10A7 is upregulated in LGG tissues and cell lines. Furthermore, SLC10A7 knockdown inhibited the proliferation and migration of LGG cells. Thus, as one of the most important TPRs, SLC10A7 is not only crucial for the maintenance of the tumor microenvironment of LGG but is also essential for the proliferation and migration of LGG. However, future *in vitro* studies are still required to elucidate the role of 14 candidate genes in the development, stemness, and immunogenicity of LGG using the LGG mouse model.

In conclusion, we identified two distinct TPR subtypes and constructed a TPR model to elucidate the characteristics of T cell proliferation in LGG and its association with immune status and prognosis. We further demonstrated the feasibility of this model by demonstrated that SLC10A was critical for the progression and migration of LGG. Thus, these findings shed light on novel immunotherapeutic strategies for LGGs.

## Data availability statement

The datasets presented in this study can be found in online repositories. The names of the repository/repositories and accession number(s) can be found in the article/**Supplementary Material**.

## Author contributions

YaL conducted the bioinformatic analysis, analyzed the data and drafted the manuscript. GP supervised and verified the experimental results. FL and YF collected and recorded the data. YuL conducted the experiments, analyzed the data, and revised the manuscript. All authors contributed to the article and approved the submitted version.

## Supplementary material

Supplementary Figure 1 The pathway enrichment analysis between subtype 2 and subtype 1. **(A-E)** Enrichment analysis of differentially expressed TPRs using the GSEA (A)/KEGG (B)/GO terms (C-E).

Supplementary Figure 2 The differential expression of TPRs in LGG samples and normal brain tissues. **(A-B)** The heatmap and volcano diagram exhibiting the differentially expressed TPRs between LGG tumors and normal tissues.

Supplementary Figure 3 The pathway enrichment analysis between the two risk groups. **(A)** The comprehensive function enrichment analysis (GO/KEGG) of differentially expressed TPRs between high-risk and low-risk groups. **(B-E)** The GSEA analysis of immune-related pathways between low-risk and high-risk groups.

Supplementary Figure 4 The differential expression of immune-related genes between high-risk and low-risk groups in the TCGA-LGG cohort, including immune stimulators **(A)**, MHC molecules **(B)**, immune inhibitors **(C)**, chemokine receptors **(D)**, and chemokines **(E)**.

Supplementary Figure 5 The relationship between TRP signature and mutation (*ATRX* and *TP53*) status. **(A-B)** The risk score between mutant (*TP53*(A) and *ATRX* (B)) and wild-type group. **(C-D)** The subgroup survival analyses of ATRX mutation and TP53 mutation between high-risk and low-risk group.
