# Supplementary figures and images for: Positive regulators of T cell functions as predictors of prognosis and microenvironment characteristics of low-grade gliomas
Source: Front Immunol. 2023 Jan 16;13:1089792. doi: 10.3389/fimmu.2022.1089792 (PMC9885161; doi:10.3389/fimmu.2022.1089792)

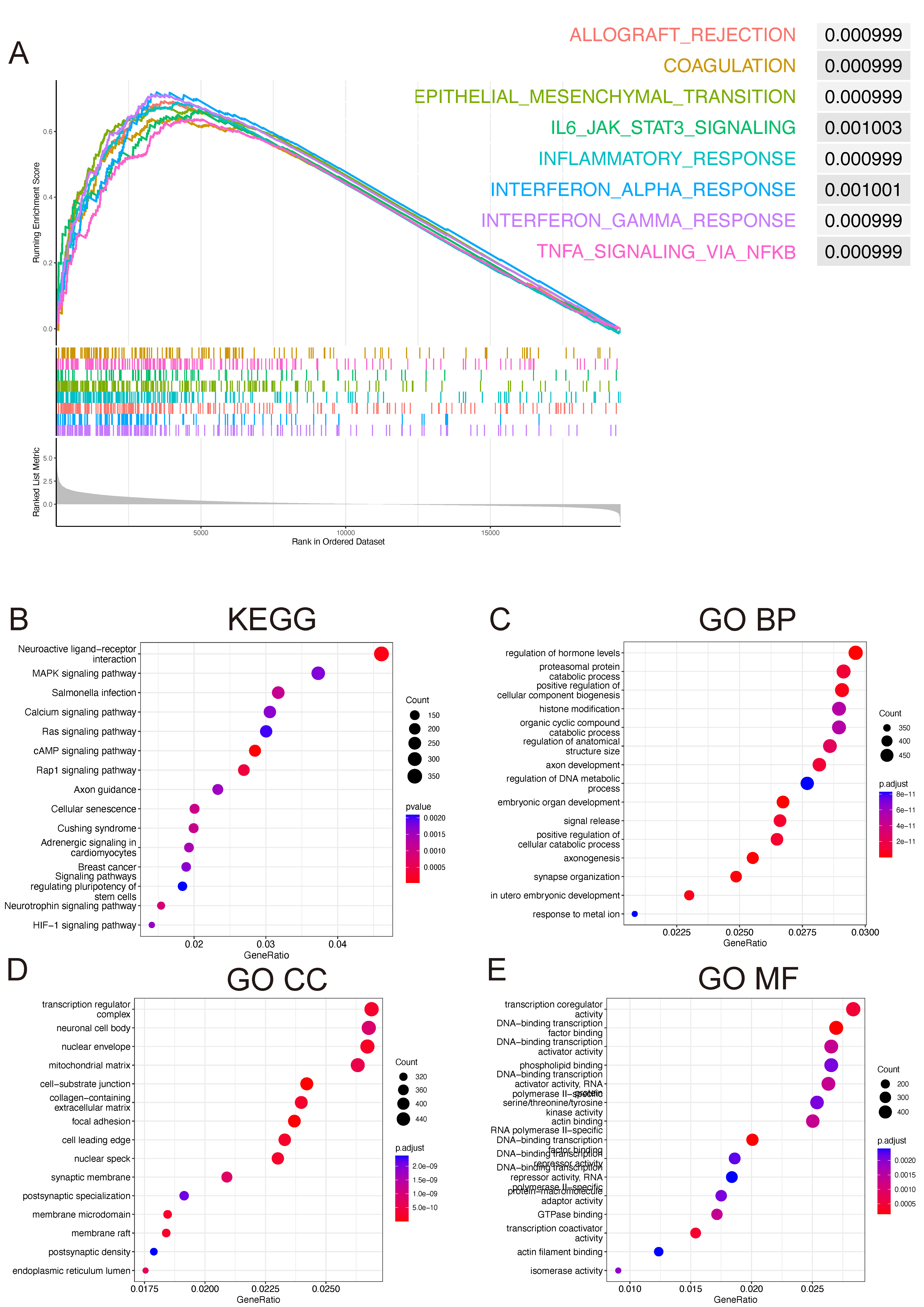

Supplement: Supplementary Figure 1 — The pathway enrichment analysis between subtype 2 and subtype 1. (A-E) Enrichment analysis of differentially expressed TPRs using the GSEA (A)/KEGG (B)/GO terms (C-E). [file Image_1.jpeg]

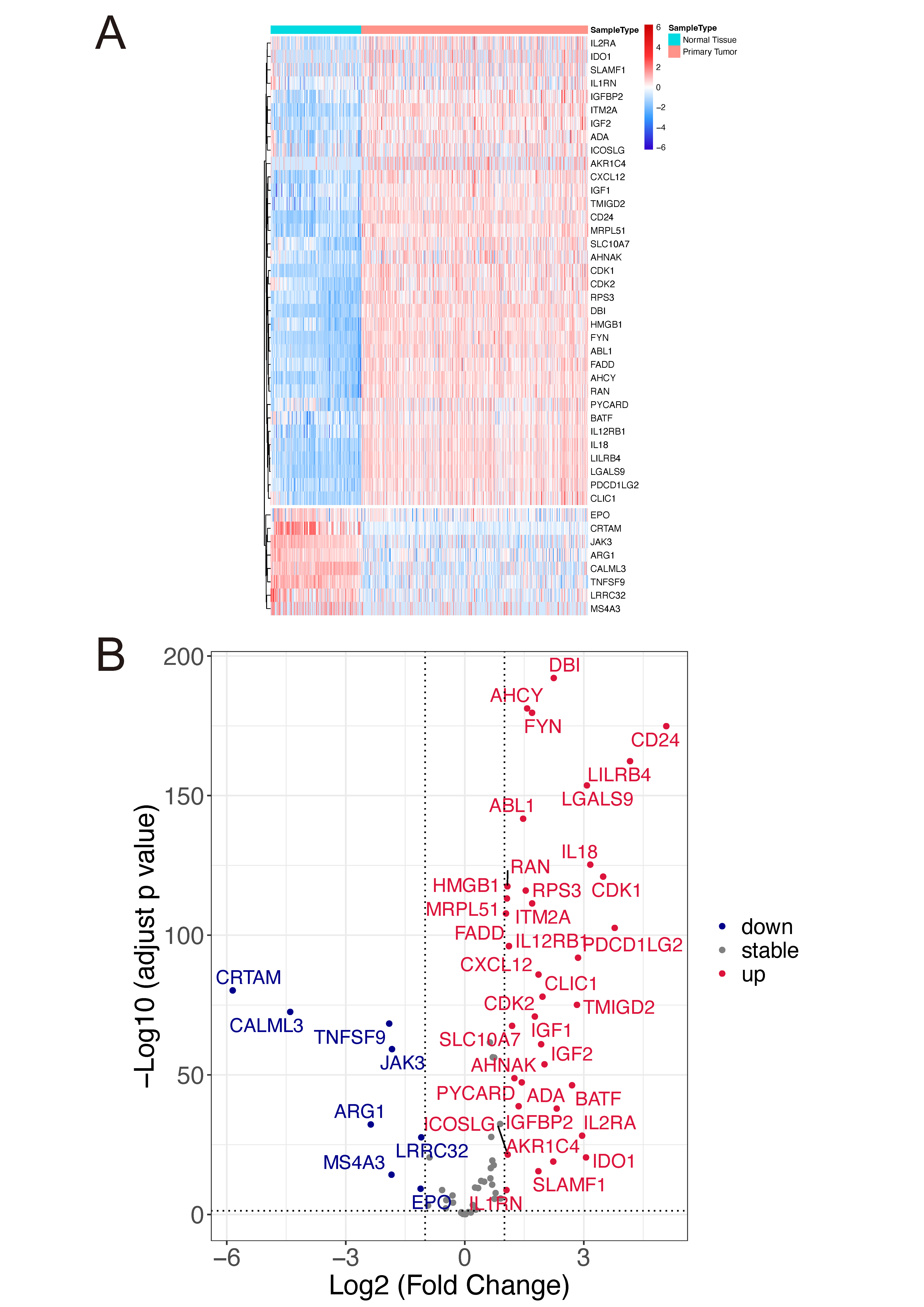

Supplement: Supplementary Figure 2 — The differential expression of TPRs in LGG samples and normal brain tissues. (A-B) The heatmap and volcano diagram exhibiting the differentially expressed TPRs between LGG tumors and normal tissues. [file Image_2.jpeg]

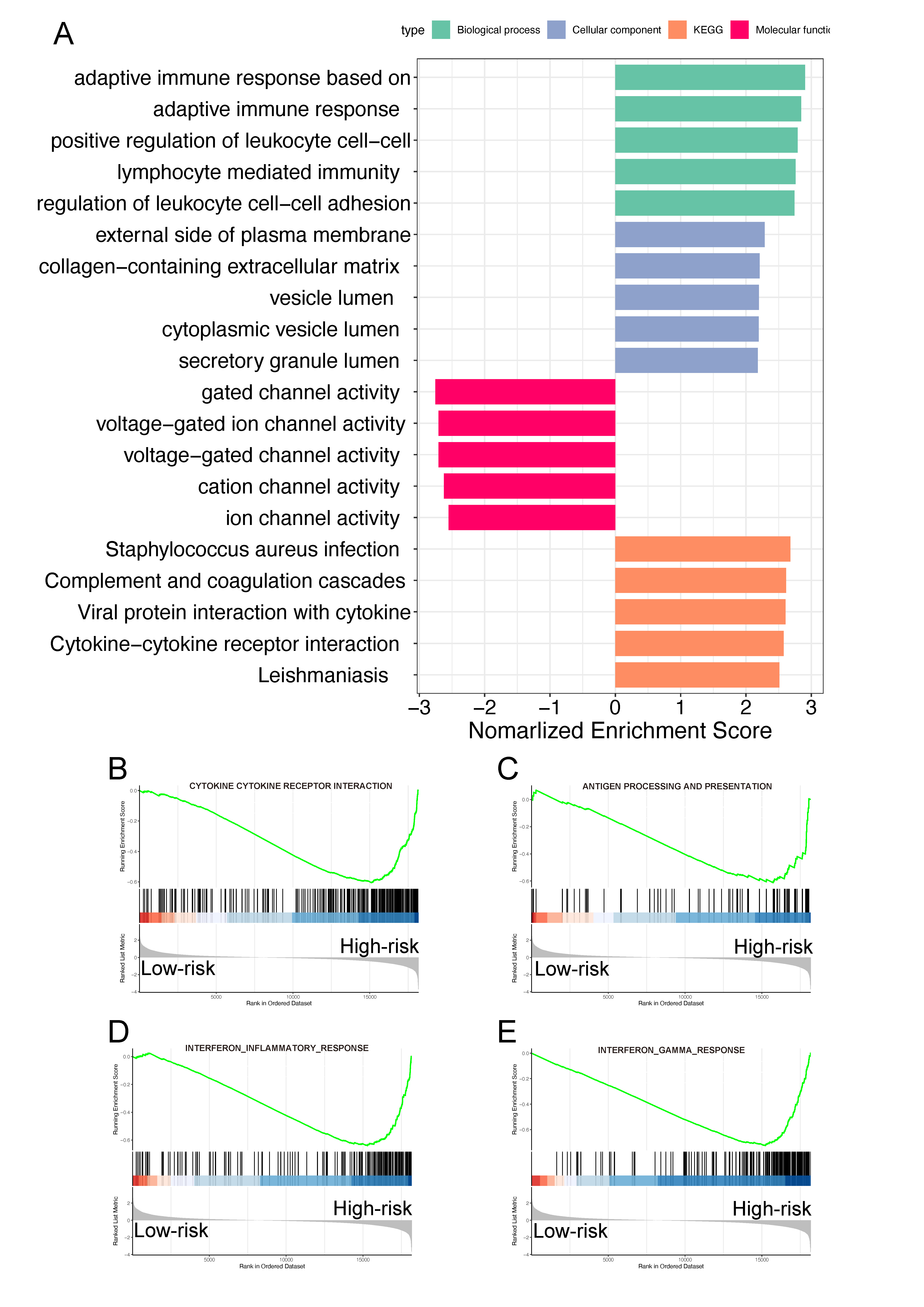

Supplement: Supplementary Figure 3 — The pathway enrichment analysis between the two risk groups. (A) The comprehensive function enrichment analysis (GO/KEGG) of differentially expressed TPRs between high-risk and low-risk groups. (B-E) The GSEA analysis of immune-related pathways between low-risk and high-risk groups. [file Image_3.jpeg]

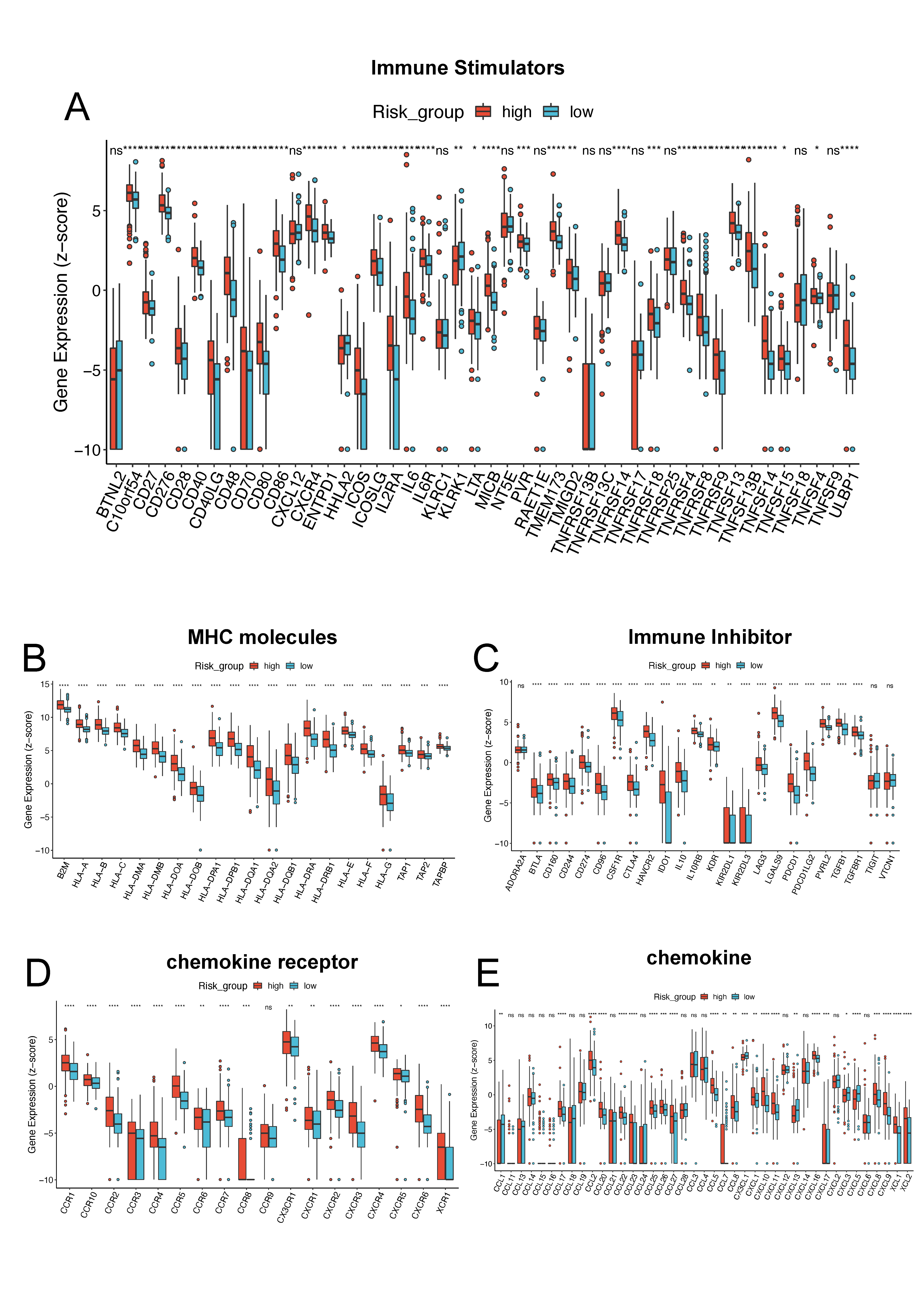

Supplement: Supplementary Figure 4 — The differential expression of immune-related genes between high-risk and low-risk groups in the TCGA-LGG cohort, including immune stimulators (A), MHC molecules (B), immune inhibitors (C), chemokine receptors (D), and chemokines (E). [file Image_4.jpeg]

**A****TP53**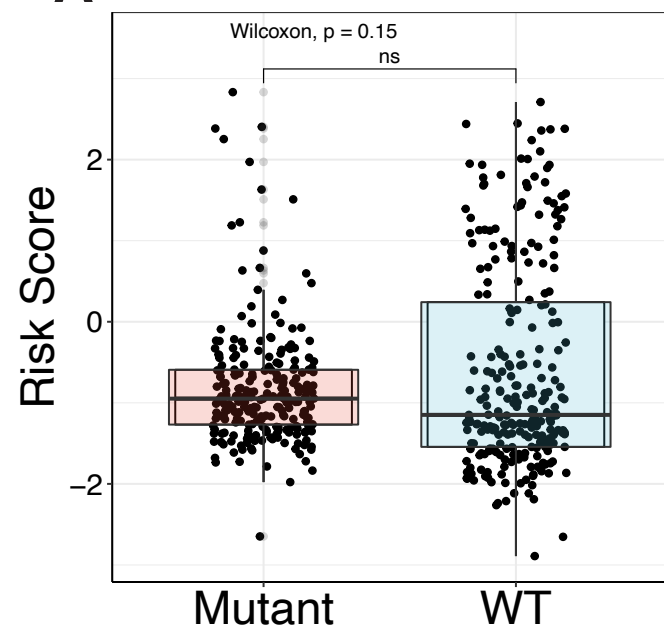**B****ATRX**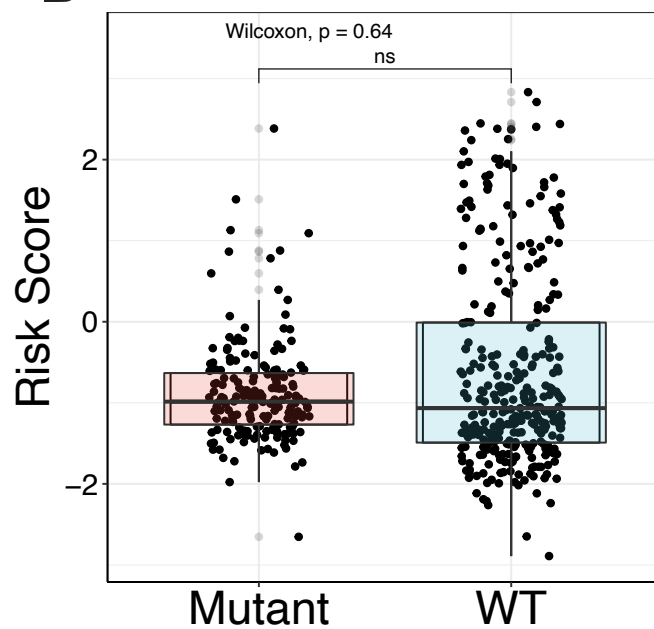**C**

Strata — High — Low

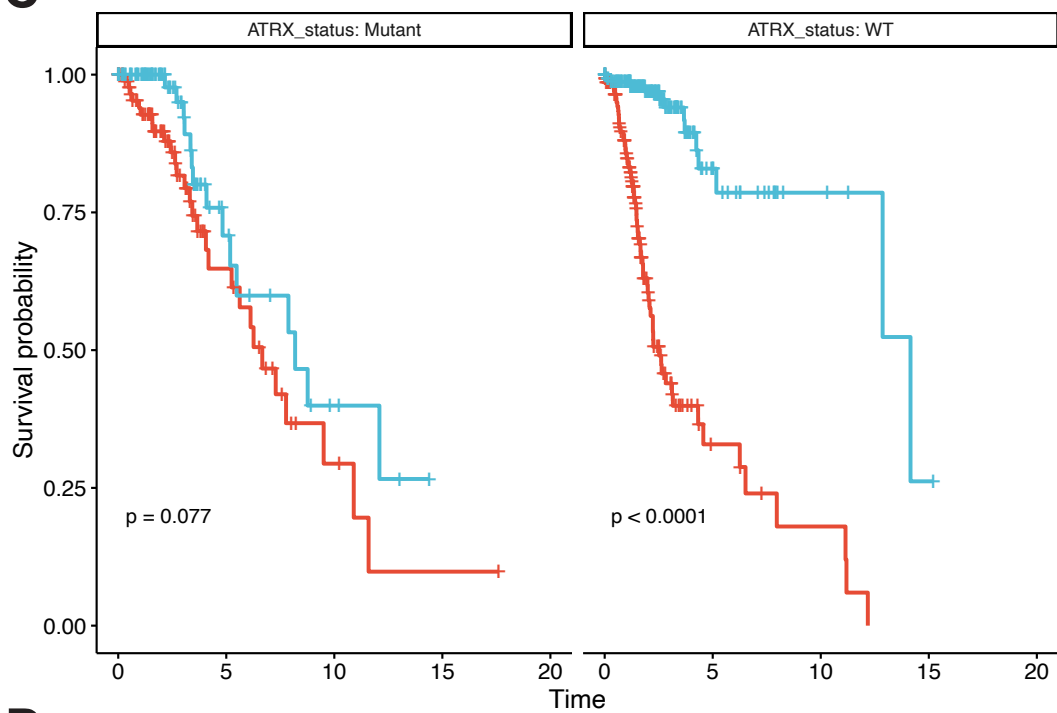**D**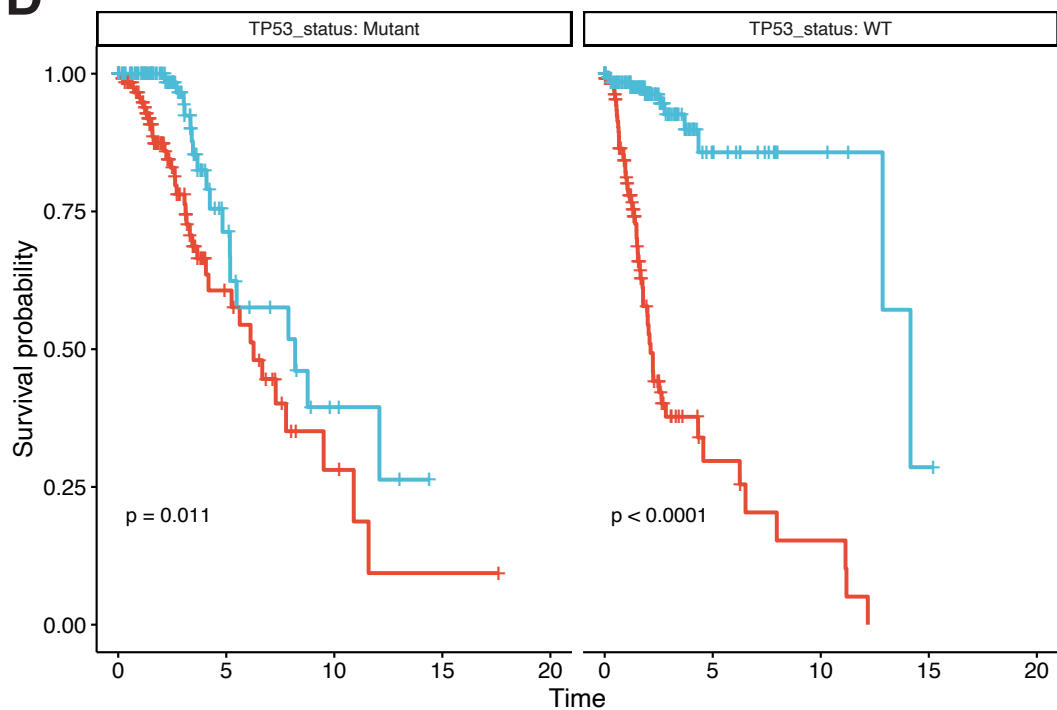

Supplement: Supplementary Figure 5 — The relationship between TRP signature and mutation (ATRX and TP53) status. (A-B) The risk score between mutant (TP53(A) and ATRX (B)) and wild-type group. (C-D) The subgroup survival analyses of ATRX mutation and TP53 mutation between high-risk and low-risk group. [file Image_5.pdf]
